# Enrichment of Secondary Wastewater Sludge for Production of Hydrogen from Crude Glycerol and Comparative Evaluation of Mono-, Co- and Mixed-Culture Systems

**DOI:** 10.3390/ijms17010092

**Published:** 2016-01-13

**Authors:** Vinayak Laxman Pachapur, Prianka Kutty, Satinder Kaur Brar, Antonio Avalos Ramirez

**Affiliations:** 1 Centre-Eau Terre Environnement, 490, Institut National de la Recherche Scientifique, Rue de la Couronne, Québec, QC G1K 9A9, Canada; vinayak.pachapur@ete.inrs.ca; 2 Department of Biotechnology, National Institute of Technology Warangal, Warangal, Telangana 506004, India; priankag1994@gmail.com; 3 Centre National en Électrochimie et en Technologies Environnementales, 2263, Avenue du Collège, Shawinigan, QC G9N 6V8, Canada; aaramirez@cnete.qc.ca

**Keywords:** crude glycerol, hydrogen, mixed-culture, secondary wastewater sludge, 1,3-propanediol

## Abstract

Anaerobic digestion using mixed-culture with broader choice of pretreatments for hydrogen (H_2_) production was investigated. Pretreatment of wastewater sludge by five methods, such as heat, acid, base, microwave and chloroform was conducted using crude glycerol (CG) as substrate. Results for heat treatment (100 °C for 15 min) showed the highest H_2_ production across the pretreatment methods with 15.18 ± 0.26 mmol/L of medium at 30 °C in absence of complex media and nutrient solution. The heat-pretreated inoculum eliminated H_2_ consuming bacteria and produced twice as much as H_2_ as compared to other pretreatment methods. The fermentation conditions, such as CG concentration (1.23 to 24 g/L), percentage of inoculum size (InS) (1.23% to 24% *v*/*v*) along with initial pH (2.98 to 8.02) was tested using central composite design (CCD) with H_2_ production as response parameter. The maximum H_2_ production of 29.43 ± 0.71 mmol/L obtained at optimum conditions of 20 g/L CG, 20% InS and pH 7. Symbiotic correlation of pH over CG and InS had a significant (*p*-value: 0.0011) contribution to H_2_ production. The mixed-culture possessed better natural acclimatization activity for degrading CG, at substrate inhibition concentration and provided efficient inoculum conditions in comparison to mono- and co-culture systems. The heat pretreatment step used across mixed-culture system is simple, cheap and industrially applicable in comparison to mono-/co-culture systems for H_2_ production.

## 1. Introduction

Biodiesel and biohydrogen are considered as renewable, efficient and carbon dioxide (CO_2_)-free fuel of choice for the future [1,2]. Biodiesel production across the world is increasing rapidly and estimated to reach 20 billion liters in 2020 due to strong government policies and incentives across the world [3,4]. About 100 kg of crude glycerol (CG) is generated as waste by-product with every ton of biodiesel produced [5]. Sustainable production and commercialization of biodiesel depends on the demand and increased utilization of CG [3]. With presence of various impurities across CG, refining for glycerin is no longer cost-effective with decreasing market value for glycerin [4,6]. Value added utilization (valorization) of waste CG into biofuels or biochemical for additional market value represents a promising route with several advantages [7,8,9]. New valorization methods for the CG are exploited as a low cost, abundant feedstock, with increased substrate conversion efficiency and decreased operating costs in comparison to other organic wastes [1,6,7].

The present hydrogen (H_2_) production techniques by physico-chemical routes are fossil fuel dependent, expensive and release CO_2_ [10]. Microbial conversion of CG to H_2_ production is an attractive approach [6,11] and has gained advantage for high energy recovery potential [8,12]. H_2_ production with utilization of waste by-product will credit CG to reduce total production costs (by 13%–14%) of biodiesel fuels [3]. The presence of various impurities in CG is the major bottleneck for the production and recovery of value-added products [13]. However, the produced H_2_ can be easily separated from the fermentation media, requiring no additional purification steps and can be directly used as fuel [14], thus reducing the downstream processing cost in comparison to value-added products, such as 1,3-propanediol (1,3-PD) and ethanol requiring production at higher concentrations to minimize costly purification steps [10].

Microbial H_2_ production using organic wastes can be divided into two steps; dark fermentation and the photo-fermentation process [15]. Dark fermentation in absence of light has advantages in terms of a simple reactor set-up, and economical increased H_2_ production rate in comparison to photo-fermentation requiring complex set-up in the presence of a light source [15,16,17]. Conversion of complex organic wastes into simple low molecular weight volatile fatty acids, H_2_ and CO_2_ during acidogenesis/acidification step of conventional anaerobic digestion is known as dark fermentation [18,19].

Anaerobic digestion has been considered economical during treatment of complex organic wastes [18], and researchers are focused on developing the acidogenesis stage of H_2_ production using mixed-culture that could be affordable and accessible [20,21]. Anaerobic digestion in the presence of mixed-culture have broader choice of organic waste feedstocks, and are easy to operate and control the growth of cultures during H_2_ production [22].

Pretreatment steps to remove H_2_ consuming bacteria that coexist in mixed-culture is a necessary step and carried out using media enrichment, heat-shock, acid, alkali, chloroform and ultrasonication techniques [21,22,23,24]. Five pretreatment methods across acid, chemical, wet heat-, dry heat-shock, freezing and thawing were carried out for cattle manure sludge [25]. A comparative study across acid, heat and chloroform treatment on sewage sludge was carried out to enrich H_2_ producing bacteria and to eliminate methane production permanently [26]. Media enrichment is the only technique explored to date for activated sludge for H_2_ production by mixed-culture using CG [1,8,12]. Highest H_2_ yield by mixed-culture using vinasse (waste of sugarcane ethanol distillation columns) resulted in 3.66 mol H_2_/mol sucrose [15] and using CG resulted in around 0.90–0.96 mol H_2_/mol glycerol [1,8]. In this study the top five commonly appearing enrichment methods, such as heat, acid, base, microwave and chloroform were conducted to increase H_2_ yield using CG.

The cattle manure sludge, reactor waste, soil types *etc.* are commonly used as seed inoculum across mixed-culture studies; in this study wastewater sludge was selected for H_2_ production. The temperature of the wastewater sludge (30 °C) at the time of collection favors the growth of microorganisms. However, the optimum temperature for H_2_ producing microorganisms is around 37 °C and around 55 °C inhibits H_2_ consuming bacteria [8]. Initial experiments at different temperatures were evaluated in this study. Optimization of fermentation parameters using statistical tools such as Central Composite Design (CCD) narrow down experimental runs, suggest the optimum parameter range and identify the dominant parameter responsible for H_2_ production in fewer runs [4,15]. The parameters needed for optimization are the substrate concentration, media supplements, endo-nutrients, inoculum size and the fermentation pH [8,12,27]. Each of these input parameters are considered as important and play a dominant effect in determining the H_2_ production [8,12]. In this study, costly media supplements and endo-nutrients concentration was omitted to decrease the process cost by eliminating the use of expensive media components. H_2_ production was carried out using minimal medium in presence of CG as substrate.

The impurities in CG derived from restaurant and meat processing waste have increased inhibition effect on pure-/co-cultures in comparison to impurities in CG derived from pure substrates [28]. The mixed-culture nullifies the inhibition effect of impurity to utilize complex CG with synergistic effects that appears advantageous over pure and co-culture system during glycerol fermentation [1,22]. Utilization of CG as substrate during glycerol fermentation for increased H_2_ yield across pure-, co- and mixed-culture system was evaluated in this study.

Glycerol fermentation follows two possible pathways (oxidative and reductive) [10]. During the oxidative pathway glycerol is converted to H_2_ and various organic acids/alcohols. In the case of the reductive pathway, glycerol is reduced into 1,3-PD production [10,29]. A complete shift of glycerol fermentation from a reductive to oxidative pathway with decreased 1,3-PD production will increase H_2_ production [5,10]. Analysis of 1,3-PD as a response factor in the central composite design (CCD) will determine the behaviour of the mixed-culture for the possible pathway during glycerol fermentation.

To date, there is disagreement on the best pretreatment methods of sludge for enriching hydrogen-producing bacteria for maximum H_2_ yield [30]. In this study, enrichment of secondary wastewater sludge using different pretreatment techniques was carried out to obtain stable consortia of mixed-culture to use CG as sole substrate for H_2_ production. To optimize the fermentation parameters, such as CG concentration, inoculum size and fermentation pH, central composite design was used along with H_2_ as response factor. The present study also deals with a comparative platform for H_2_ production using pure-, co- and mixed-culture system with CG as substrate.

## 2. Results and Discussion

### 2.1. Hydrogen Production Using Different Pretreatment Methods

The pretreatment methods tested in this study for wastewater sludge for the inoculum enrichment for H_2_ production are presented in the Table 1. The heat treatment resulted in increased production (15.18 ± 0.26 mmol/L) in comparison to other methods using CG as substrate. The production using heat treatment at 30 °C (15.18 ± 0.26 mmol/L) was highest in comparison to 55 °C (4.57 ± 0.53 mmol/L) and 37 °C (12.76 ± 0.50 mmol/L). The same set-up of heat treatment was tested at 25 °C, however there was no increase in H_2_ production in comparison to 30 °C (data not shown). The fermentation temperature optimum for H_2_ production using pretreated wastewater sludge was highest at 30 °C in comparison to other temperatures as seen in Table 1. The highest H_2_ production of 15.18 ± 0.26 mmol/L-of medium was obtained for the heat pretreatment method at 30 °C. The lowest H_2_ production of 1.62 ± 0.91 mmol/L was observed in the case of chloroform treatment. H_2_ production increased with a decrease in the incubation temperature across all the pretreatment methods. The highest production of 15.18 ± 0.26 mmol/L in case of heat pretreatment of wastewater sludge at 30 °C was shortlisted for further optimization experiments using CCD.

**Table 1:** Hydrogen production (mmol/L) for different pretreatment methods across different fermentation temperature conditions.

Pretreatment Methods	Hydrogen Production (mmol/L) across Different Temperature Set-up		
	55 °C	37 °C	30 °C
Heat	4.57 ± 0.53	12.76 ± 0.50	15.18 ± 0.26
Acid	1.94 ± 0.22	4.40 ± 0.51	7.05 ± 0.83
Alkali	4.22 ± 0.49	4.67 ± 0.59	10.02 ± 0.22
Microwave	3.98 ± 0.46	4.22 ± 0.49	6.09 ± 0.71
Chloroform	1.62 ± 0.91	2.71 ± 0.31	9.91 ± 0.68

### 2.2. Hydrogen Production during Optimization Studies

The H_2_ production across different CG concentrations, inoculum sizes and varying pH is represented in the Table 2. H_2_ production ranged from 5.11 ± 0.71 (obtained at CG: 20, InS: 6, pH: 4) to a maximum of 29.43 ± 0.71 mmol/L (obtained at CG: 20, InS: 20, pH: 7).

**Table 2:** The experimental runs of central composite design across crude glycerol concentration (g/L), different inoculum size (%), varying pH and experimental runs in terms of hydrogen production (mmol/L) and 1,3-PD concentration (g/L).

Run	Crude Glycerol (g/L)	Inoculum Size (%)	pH	Hydrogen Production (mmol/L)	1,3-PD (g/L)
1	20	6	4	5.11 ± 0.71	0.77 ± 0.04
2	6	6	7	17.62 ± 0.84	3.15 ± 0.26
3	13	13	5.5	17.28 ± 0.40	1.57 ± 0.23
4	13	13	5.5	18.81 ± 0.83	1.45 ± 0.30
5	6	6	4	5.21 ± 0.55	1.56 ± 0..56
6	1.23	13	5.5	9.86 ± 0.88	0.53 ± 0.62
7	20	20	7	29.43 ± 0.71	5.12 ± 0.59
8	13	13	5.5	17.28 ± 0.52	1.68 ± 0.76
9	20	6	7	21.84 ± 0.78	6.02 ± 0.56
10	6	20	4	8.06 ± 0.89	2.69 ± 0.75
11	20	20	4	8.41 ± 0.21	1.67 ± 0.62
12	13	13	5.5	18.44 ± 0.19	1.55 ± 0.66
13	13	13	2.98	8.51 ± 0.71	1.28 ± 0.58
14	24	13	5.5	10.51 ± 0.76	2.00 ± 0.67
15	13	13	5.5	19.28 ± 0.71	1.72 ± 0.77
16	13	1.23	5.5	9.94 ± 0.10	0.96 ± 0.64
17	6	20	7	17.12 ± 0.25	3.07 ± 0.66
18	13	24	5.5	12.26 ± 0.92	1.84 ± 0.73
19	13	13	8.02	14.55 ± 0.54	6.06 ± 0.59
20	13	13	5.5	18.68 ± 0.21	1.22 ± 0.62

The response surface quadratic model with *p*-value of 0.028 was significant, and pH with *p*-value of 0.0011 had a significant effect on H_2_ production. The model ANOVA results for H_2_ production are represented in the Table 3. The significant *p*-value of 0.0011 indicated the linear dominance of pH parameter on H_2_ production in comparison to CG and inoculum size.

**Table 3:** The model ANOVA results for hydrogen production and 1,3-PD concentration.

Source	*p*-Value	
	Hydrogen	1,3-Propanediol
Model significant	0.028	<0.0001
A-crude glycerol	0.2701	0.0147
B-inoculum size	0.289	0.2138
C-pH	0.0011	<0.0001
AB	0.4828	0.4875
AC	0.1949	0.0009
BC	0.9376	0.064
A2	0.0824	0.5269
B2	0.1334	0.3378
C2	0.166	<0.0001

The model equation that best represented the fitting data is shown below in (Equation (1)):

(1) Hydrogen = +18.18 + 1.31 × CG + 1.26 × InS + 5.08 × pH + 1.07 × CG × InS + 2.04 × CG × pH + 0.12 × InS × pH − 2.11 × CG × CG − 1.78 × InS × InS − 1.63 × pH × pH

**Figure 1:**
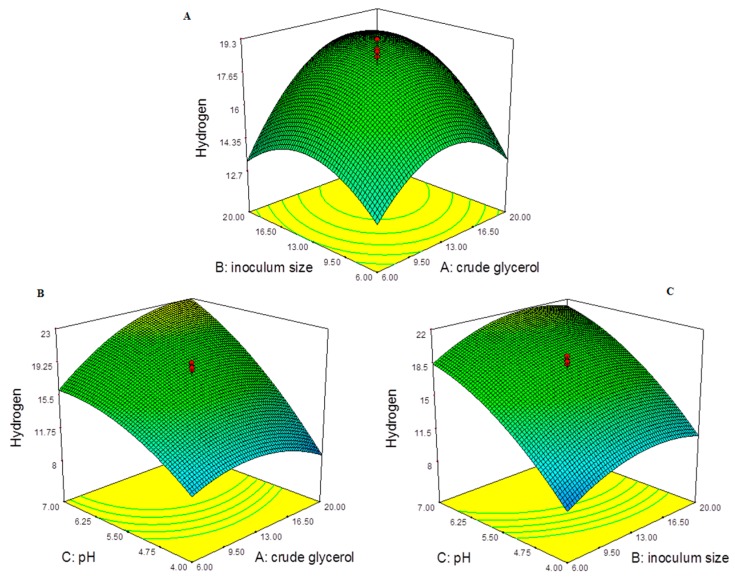
Hydrogen production (mmol/L) response across the input parameters: (**A**) across crude glycerol (g/L) and inoculum size (%); (**B**) across crude glycerol (g/L) and (**C**) inoculum size (%) and pH.

Across Equation (1), pH (5.08) had a positive value and was higher in comparison to other input parameters, indicating the influence of pH to be greater at higher value with dominating effect on H_2_ production. The H_2_ production response across the input parameters: CG, inoculum size and pH using response surface curve are represented in the Figure 1. At minimum concentration of CG with increasing inoculum size, the H_2_ production increased and decreased as seen in Figure 1A. The H_2_ production at minimum concentration of CG (6 g/L) and InS (6%) for run:2 and 5, varied from 5.21 ± 0.55 to 17.62 ± 0.84 mmol/L. In case of increased inoculum size of 20% at minimum concentration of CG (6 g/L), the H_2_ varied from 8.06 ± 0.89 to 17.12 ± 0.25 mmol/L. At maximum concentration of CG with increasing inoculum size, the H_2_ production increased. This is seen upon run:1 (CG: 20, InS: 6) with 5.11 ± 0.71 mmol/L increased to 29.43 ± 0.71 mmol/L of H_2_ production for run:7 at (CG: 20, InS: 20). The effect of increase in InS at maximum CG concentration had increasing effect on H_2_ production as seen in the Figure 1A. The H_2_ production at minimum concentration of CG (6 g/L) increased with increase in the pH as seen in the Figure 1B. This is seen in run:5 (CG: 6, pH: 4) with 5.21 ± 0.55 mmol/L increased to 17.12 ± 0.25 mmol/L of H_2_ production for run:17 at (CG: 6, pH: 7). This was even true at maximum concentration of CG for run:11 (CG: 20, pH: 4) with 8.41 ± 0.21 mmol/L increased to 29.43 ± 0.71 mmol/L for the run:7 (CG: 20, pH: 7). The effect of increase in pH across CG concentrations had a similar effect with increase in H_2_ production till pH nearing to optimum (pH 7.0) as seen in the Figure 1B. The relation of H_2_ production between CG and pH was also similar with pH and InS as seen with the response curve in the Figure 1C. For run:1 (InS: 6, pH: 4) with 5.11 ± 0.71 mmol/L increased to 21.84 ± 0.78 mmol/L of H_2_ production for run:9 at (InS: 6, pH: 7). Similar increase was true at maximum concentration of CG for run:11 (InS: 20, pH: 4) with 8.41 ± 0.21 mmol/L increased to 29.43 ± 0.71 mmol/L for the run:7 (InS: 20, pH: 7). The effect of increase in pH across inoculum size had similar effect with increase in H_2_ production till pH nearing to optimum (pH 7.0) as seen in the Figure 1C. The optimum fermentation conditions are dependent on substrate concentration, working pH and inoculum size for increased H_2_ production [2,5]. The fermentation pH had the dominant effect across the other input parameters and at neutral pH resulted in maximum H_2_ production at maximum CG (20 g/L) with 20% inoculum size.

### 2.3. 1,3-Propanediol Production during Optimization Studies

The response of 1,3-PD production across different CG concentrations, inoculum sizes and varying pH is represented in Table 2. 1,3-PD production ranged from minimum of 0.53 ± 0.62 (run:6, CG: 1.23, InS: 13, pH: 5.5) to a maximum of 6.06 ± 0.59 g/L (run:19, CG: 13, InS: 13, pH: 8.02). The response surface quadratic model with *p*-value of <0.0001 was significant. The *p*-value of CG (0.0147) and pH (<0.0001) both had a significant effect on 1,3-PD production. The model equation that best represented the fitting data has been shown below in Equation (2):

(2) 1,3-Propanedion = +1.50 + 0.41 × CG + 0.18 × InS + 1.37 × pH − 0.13 × CG × InS + 0.84 × CG × pH − 0.38 × InS × pH + 0.08 × CG × CG + 0.14 × InS × InS + 0.94 × pH × pH

Across Equation (2), akin to H_2_ production, the coefficient of pH (1.37) had a positive value and was higher in comparison to other input parameters. The significant *p*-value of <0.0011 indicated the linear dominance of pH along with CG (0.0147) parameter on 1,3-PD production as seen in Table 3.

**Figure 2:**
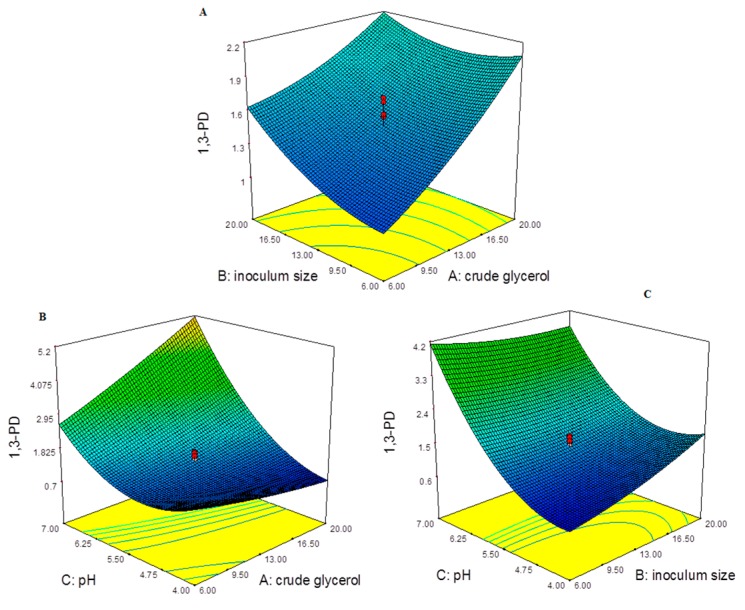
1,3-PD production (g/L) response across the input parameters: (**A**) crude glycerol (g/L) and inoculum size (%); (**B**) across crude glycerol (g/L) and (**C**) inoculum size (%) and pH.

The 1,3-PD production response across the input parameters: CG, inoculum size and pH using response surface curve are represented in Figure 2. At maximum concentration of CG with increasing inoculum size, 1,3-PD production was less than maximum response (6.06 g/L) as seen in Figure 2A. The 1,3-PD production at maximum concentration of CG (20 g/L) with increasing InS from 6%–20% for run:1 and 11, ranged between 0.77 ± 0.04 to 1.67 ± 0.62 g/L. The response of 1,3-PD increased with increase in CG concentration at every interval of InS as seen in the Figure 2A. The 1,3-PD production at maximum concentration of CG (20 g/L) increased with increase in the fermentation pH as seen in the Figure 2B. This is seen in run:1 (CG: 6, pH: 4) with 0.77 ± 0.04 g/L increased to 5.12 ± 0.59 and 6.02 ± 0.56 g/L for run:7 and 9 at (CG: 20, pH: 7). The production of 1,3-PD reached maximum in the pH range (7–8), which was also dependent on the CG concentration. The relation of 1,3-PD production between CG and pH was also similar with pH and InS as seen with the response curve in the Figure 2C. In the case of run:10 and 11 (InS: 20, pH: 4) the range was from 1.67 ± 0.62 to 2.69 ± 0.75 g/L and increased in a range from 3.07 ± 0.66 to 5.12 ± 0.59 g/L of 1,3-PD production for run:7 and 17 at (InS: 20, pH: 7). The production of 1,3-PD reached maximum in the pH range (7–8), which was also dependent on the InS. The decreased production of 1,3-PD during glycerol fermentation increased the H_2_ production [2,10]. The maximum H_2_ production of 29.43 ± 0.71 mmol/L was observed at 5.12 ± 0.59 g/L of 1,3-PD production, which was lower in comparison to maximum production of 1,3-PD (6.06 ± 0.59 g/L) resulting in decreased H_2_ production with 14.55 ± 0.54 mmol/L.

Enrichment methods using acid, chemical, wet heat-, dry heat-shock, freezing and thawing was carried out for cattle manure sludge, and increased H_2_ production using acid pretreatment was 1.9–9.8 times greater compared to control sludge [25]. A comparative study was made across acid, heat and chloroform treatment on sewage sludge during immobilized H_2_ production in a mixed-culture under anaerobic conditions. Chloroform treatment inhibited hydrogen consuming bacteria, avoided fast conversion of H_2_ to acetic acid and repeated culture showed H_2_ production till 15 days [26]. Heat pretreatment was the best among other pretreatments (acid and base) for enriching H_2_ producing bacteria from anaerobic sludge [31]. The increased H_2_ production depends on the source of the seed sludge and the pretreatment method. Heat treatment is a simple, effective and practical method in comparison to other methods for enriching H_2_-producing bacteria from different seed source [30,31,32,33]. The effect of heat pretreatment depends on the seed inoculum to eliminate H_2_ consumers and also on the kind of substrate used during fermentation [32]. In this study, the heat-pretreated inoculum possessed higher natural acclimatization activity for degrading CG and produced twice as much as H_2_ in comparison to other pretreatment methods.

The results obtained across pretreatment methods over different fermentation temperatures, helped to narrow down the input parameters for the CCD model. The input parameters using CG concentration ranged from 1.23 to 24 g/L, inoculum size from 1.23% to 24% and pH from 2.98 to 8.02, respectively along with responses of H_2_ and 1,3-PD production represented in the Table 2. The central point from CCD model at CG (13 g/L), InS (13%) and pH (5.5) resulted in the production of H_2_ reaching only 18.81 ± 0.83 mmol/L in comparison to 15.18 ± 0.26 at CG (10 g/L). The increase in concentration of CG with increasing inoculum size increased H_2_ production, however the effect of initial pH was dominant and had a significant effect (*p*-value: 0.0011) in increasing H_2_ production. At maximum concentration of CG (20 g/L) for run:1 and 11, with increase in inoculum size from six to 20% resulted in marginal H_2_ production from 5.11 ± 0.71 to 8.41 ± 0.21 mmol/L. However, at the same values of CG and InS, when the pH was adjusted from 4 to 7, the H_2_ production increased with 29.43 ± 0.71 mmol/L. Further increase in the initial pH to 8.02 at CG (13 g/L) and InS (13%) resulted in decreased H_2_ production reaching only 14.55 ± 0.54 mmol/L. Anaerobic digestion for H_2_ production depends on growth limitation of methanogens, performance of H_2_ production and shift from acidogenesis to solventogenesis depends on the pH condition [8]. The optimum pH for H_2_ production is effective, when the methanogenic bacteria get repressed at pH 5.5–6 and also with heat-shock pretreatment (100 °C) [33]. The conditions of initial pH: 7 in presence of CG (20 g/L) and InS (20%) was optimum for the growth of H_2_ producers in mixed-culture, which resulted in increased H_2_ production reaching 29.43 ± 0.71 mmol/L.

The H_2_ yield comparison along with seed inoculum, experimental condition, and type of design used across mixed-culture studies is represented in Table 4. The results obtained with hot air oven pretreatment for anaerobic granule sludge using CG (22.19 g/L) in presence of endo-nutrient (2.89 mL/L) resulted in H_2_ production of 1.37 mmol/L h [27]. The conversion of CG into H_2_ with heat-treated mixed-culture at substrate concentration of 3 g/L in presence of nutrient solutions resulted in 0.31 mol H_2_/mol glycerol [32]. Enrichment of activated sludge using minimal medium in presence of CG (15 g/L) resulted in an H_2_ yield ranging from 0.66 to 0.96 mol H_2_/mol of glycerol [1,8]. The highest H_2_ yield of 1.41 mol/mol of glycerol was obtained from enrichment of activated sludge using complex modified HM 100 medium using CG (1 g/L) [12]. The optimal condition in this study resulted in H_2_ production rate of around 1.23 mmol/L h and with 72% of substrate utilization at H_2_ yield of 0.82 mol/mol of glycerol. The results obtained in this study are slightly higher in presence of minimal medium at 20 g/L, and is comparable to studies (as seen in Table 4) using complex medium, nutrient solutions, and working at very low CG concentrations. The heat treatment method used in this study is simple, low cost and industrially applicable in comparison to using costly medium enrichment pretreatment methods. This study provides dual environment benefits with wastewater treatment along with utilization of CG into generation of H_2_ for possible domestic renewable energy source for the biodiesel industry.

**Table 4:** Hydrogen yield comparison across mixed-culture studies using crude glycerol as substrate.

Seed Inoculum	Pretreatment	Substrate	Experimental Condition	Experimental Design	H_2_ Yield (mol H_2_/mol glycerol)	Ref.
Anaerobic granule from an upflow anaerobic sludge blanket (UASB) reactor	105 °C for 2 h in hot-air oven	CG derived from fried chicken oil waste	CG at 22.19 g/L, supplement with endo-nutrients, initial pH 5.5 at 35 °C with 150 rpm	CCD on CG, sludge and endo-nutrient concentration	0.30	[27]
Wheat soil	105 °C for 2 h	CG derived from transesterification of soybean oil	CG at 3 g/L, supplement with nutrient solution, initial pH 6.2 at 30 °C	No design	0.31	[32]
Wastewater sludge	Media enrichment	CG derived from transesterification of rapeseed, sunflower and soy	CG at 15 g/L along with Minimal medium to dilute CG with pH 6.8 at 37 °C with 120 rpm	Plackett-Burman on CG concentration, temperature and initial pH	0.66 to 0.96	[1,8]
Activated sludge	Media enrichment	CG derived from transesterification of canola oil and restaurant fats	CG at 1 g/L along with Modified HM 100 medium to dilute CG with pH 6.5 at 40 °C with 120 rpm	Plackett-Burman with five independent variables	1.41	[12]
Wastewater sludge	100 °C for 15 min in an Isotemp Standard Lab Ovens	CG derived from transesterification of meat processing plants and used grease from restaurants	CG at 20 g/L along with Modified basal medium to dilute CG with pH 6.5 at 37 °C with 150 rpm	CCD on CG concentration, inoculum size and fermentation pH	0.82	This study

### 2.4. Hydrogen Production across Pure-, Co- and Mixed-Culture System

To determine CG substrate inhibition effect at higher concentration, H_2_ production across the mono-/co- and mixed-culture systems was carried out. The co-culture system of two defined microorganisms possesses increased substrate inhibition effect in comparison to single pure culture [4,11]. Two strains *Enterobacter aerogenes and Clostridium butyricum*, commonly used for H_2_ production using CG [13] were selected along with mixed-culture. The optimum condition for mixed-culture (CG: 20 g/L, InS: 20%) was carried out using mono- (*C. butyricum*) and co-culture system comprising (*E. aerogenes and C. butyricum*) for H_2_ production. The comparison across mono-, co- and mixed-culture system for the microorganisms used, growth media, incubation condition, incubation time along with cumulative H_2_ production and yield is presented in Table 5.

**Table 5:** Comparison of pure-, co- and mixed-culture hydrogen production processes in terms of inoculum development, hydrogen yield and by-product (1,3-PD) concentration.

Hydrogen Production Steps	Hydrogen Production System		
	Mono-Culture	Co-Culture	Mixed-Culture
Microorganisms used	*Clostridium butyricum*	*Enterobacter aerogenes* + *Clostridium butyricum*	Seed wastewater sludge
Growth media	Glucose, casein peptone, KH_2_PO_4_, MgSO_4_·7H_2_O, yeast extract and l-cysteine	Glucose, casein peptone, KH_2_PO_4_, MgSO_4_·7H_2_O, yeast extract and l-cysteine	Without growth media
Incubation conditions	37 °C at 18 h	37 °C at 18 h	100 °C for 15 min
Incubation time	18 h	18 h	15 min
Cumulative Hydrogen production (mmol/L)	13.92 ± 0.62	24.85 ± 0.92	29.43 ± 0.71
Hydrogen Yield (mol H_2_/mol glycerol)	0.39	0.69	0.82
1,3-PD concentration (g/L)	6.54 ± 0.38	6.01 ± 0.62	5.12 ± 0.59

The media pH conditions for mono- and co-culture was around 6.5, which is optimum for H_2_ production [2]. The cumulative H_2_ production (mmol/L) for mono- (13.92 ± 0.62) and co-culture system (24.85 ± 0.92) was lower in comparison to mixed-culture system (29.43 ± 0.71) and was also true in case of H_2_ yield (as seen in Table 5). Cost distribution in case of pure culture with techno-economic analysis using CG by Saurabh *et al.*, suggested alternative options for reduction of process cost [14]. One such alternative option can be use of mixed-culture system over pure- and co-culture systems to minimize the process cost in terms of growth media and incubation conditions. The complex growth media components in case of pure culture require glucose, casein peptone, KH_2_PO_4_, MgSO_4_·7H_2_O, yeast extract and l-cysteine. However, using a heat pretreatment approach for selecting H_2_ producing organisms requires no growth media components in comparison to enrichment techniques with complex modified HM 100 medium used [12]. The incubation time for pure-/co-culture system varies from 12–18 h depending upon the microorganism selected and is also similar to time required during enrichment technique. However, in the case of the mixed-culture, the heat pretreatment option can be carried out in only 15 min. The mixed-culture system in comparison to mono- and co-culture system in terms of growth media, incubation condition and time holds an additional advantage along with increased H_2_ production. In addition to increased H_2_ production, the mixed-culture also possesses the ability to work at substrate inhibitor concentration, which determines glycerol degrading syntrophic H_2_ producing microorganisms. The consortium of mixed-culture obtained from the wastewater sludge was able to produce maximum H_2_, even at substrate inhibition concentration (20 g/L). The increase in concentration from 15 to 20 g/L for *E. aerogenes* and *C. butyricum* during mono- and co-culture studies resulted in decreased H_2_ production, indicating substrate inhibition concentration at 20 g/L [2,5,10]. The wastewater sludge is the largest depositor of different kinds of microorganisms, possessing increased substrate inhibition along with increased glycerol degrading ability. The pure- and co-culture at substrate inhibition concentration (20 g/L) tends to produce 1,3-PD at higher concentration (6.54 ± 0.38 and 6.01 ± 0.62 g/L) with decreased H_2_ production (as seen in Table 5). The substrate inhibition concentration on pure- and co-culture shifts the metabolic pathway towards reductive pathway for 1,3-PD production instead of H_2_ production through the oxidative pathway. The higher the concentration of CG in the fermentation media, the higher is the production of 1,3-PD using pure- and co-culture systems (as seen in Table 5) [2,10]. The mixed-culture overcomes the substrate inhibition concentration of CG with increased H_2_ yield in comparison to pure- and co-culture system as seen from the Table 5. The microbial community analysis after the heat pretreatment belonged mostly to *Clostridium* family, and showed dominance over non-hydrogen producing microorganism [32]. However, to understand the population dynamics of mixed-culture system and its ability to work at higher CG concentration in the absence of complex media components are investigations for the future.

## 3. Materials and Methods

### 3.1. Crude Glycerol as Substrate

CG was supplied by Rothsay^®^ (Winnipeg, MB, Canada) that uses inedible fat containing waste from meat processing plants and used grease from restaurants for biodiesel production [5]. The CG contained (*w*/*w*): up to 23.6% of glycerol, 35.9% carbon and 3.2% nitrogen, 3.06% ash, 5.75% moisture and 67.56% matter organic non-glycerol [2]. This is the first time that CG derived from the processing of animal fat and restaurant waste was used for anaerobic digestion for H_2_ production using mixed-culture.

### 3.2. Seed Inoculum

The wastewater sludge was collected from Quebec Urban Community (QUC) wastewater treatment plant (WWTP) (Quebec, QC, Canada). The secondary sludge sample was collected in pre-cleaned high-density polyethylene (HDPE) containers and stored at 4 ± 1 °C, until further use. The characteristics of secondary sludge comprised in (g/L): total solids (TS) (9.15 ± 0.13), suspended solids (SS) (7.22 ± 0.35), total chemical oxygen demand (TCOD) (6.11 ± 1.4), total organic carbon (TOC) (411.82 ± 0.66), ammonia-nitrogen (0.21 ± 0.01) and pH (6.14 ± 0.41) as analyzed by [34].

### 3.3. Evaluation of Different Pretreatment Methods for Preparation of Seed Inoculum

Different pretreatment techniques for wastewater sludge to enhance and optimize the H_2_ production efficiency using CG as substrate were investigated. The top five pretreatment techniques, such as heat, acid, alkali, microwave, and chloroform use on wastewater to inhibit the H_2_ consuming bacteria and screen H_2_ producing mixed-culture were selected.

The pretreatment conditions employed were as follows: (a) Heat pretreatment (heat-shock): 50 mL of wastewater sludge was transferred into a 150 mL serum bottle, sparged with N_2_ gas for 4 min to create anaerobic condition, sealed with pre-inserted septa (Headspace 20 mm Crimp Seals with Septa, Thermo Scientific™, Pittsburgh, PA, USA) kept at 100 °C for 15 min in an Isotemp Standard Lab Ovens (Fisher Scientific™, Pittsburgh, PA, USA) and later cooled prior to inoculation for H_2_ production [35]; (b) Acid pretreatment: The pH of wastewater sludge (50 mL) was adjusted to 3.0 using 0.1 M HCl, kept at room temperature for 24 h, later pH was re-adjusted to 6.5 (working pH) using 0.1 M NaOH, transferred to serum bottle, sealed and sparged with N_2_ gas, prior to use [35]; (c) Alkali pretreatment: Similar to preparation steps of acid treatment, pH of wastewater sludge was adjusted to 12 using 0.1 M NaOH at room temperature 24 h and re-adjusted to working pH 6.5 [35]; (d) Microwave pretreatment: The wastewater sludge of around 50 mL (2 × 25 mL) was put in the microwave (MARS microwave extractor, CEM Corporation, Matthews, NC, USA) at conditions pressure (120 psi) for 2 min at 560–600 W maintained at 90 °C, the treated wastewater was collected, sparged and sealed prior to use [36]; (e) Chloroform pretreatment: The wastewater sludge (50 mL) was mixed with chloroform (0.05% *v*/*v*), transferred to serum bottle, incubated for 24 h, sparged and sealed prior to inoculation [37].

### 3.4. Hydrogen Production Using Modified Basal Media

A fermentation media containing 1% (*w/v*) CG, 2% casein peptone, 0.2% KH_2_PO_4_, 0.05% MgSO_4_·7H_2_O and 0.05% yeast extract was maintained at a pH = 6.5 and transferred to serum bottles with a working volume of 50 mL. The headspace of the bottles was purged with pure N_2_ gas for 4 min to create anaerobic conditions and later sealed with pre-inserted septa (headspace 20 mm crimp seals with septa, Thermo Scientific™) followed by sterilization in an autoclave (Tuttnauer 3870-Heidolph) [5]. The pretreated sludge with 5% (*v/v*) inoculum size was transferred into the culture broth using a sterile syringe (All-Plastic Norm-Ject™ Syringes, Thermo Scientific™) along with control experiments without any pretreatment of sludge were performed simultaneously. The serum bottles were incubated in an orbital incubator shaker (INFORS HT-multitron standard) at 150 rpm at different temperatures (30, 37 and 55 °C) for five days. All batch experiments in the study were performed in triplicates, presented values are the average of triplicates and error bars represent the standard deviation (±) values.

### 3.5. Investigating Process Parameters Using Statistical Model

The best pretreatment method resulting in increased H_2_ production was later selected for optimization experiments. Central composite design (CCD) was used to investigate the effects of substrate (CG) concentration (g/L), inoculum size (InS) (%) and fermentation pH using Design-Expert 7 software (Stat-Ease Inc., Minneapolis, MN, USA), which resulted in 20 set of experiments. The central composite design matrix comprising of varied CG concentration along with different inoculum size and pH is given in Table 2. Across the 20 experiments, the central point of the model with CG (13 g/L), InS (13%) and pH (5.5) appeared 6 times.

Each set of experiment was performed at exact CG concentration along with media supplement (casein polypeptone, KH_2_PO_4_, yeast extract and MgSO_4_·7H_2_O) was mixed in distilled water to make-up the required volume (final volume (50 mL) minus inoculum size). The required volume was pH adjusted, transferred to serum bottle, purged, sealed and sterilized. After inoculation, serum bottles were incubated in an orbital shaker at 150 rpm for 5 days at 30 °C. Each experimental run was carried out in triplicates. The gas sample (1 mL) for every 24 h was collected from the headspace using a gas tight syringe in vacuumed sample vials for H_2_ analysis by gas chromatography (GC). The aqueous sample at the end of fermentation was analyzed for glycerol and end 1,3-PD concentration by GC, as described later.

In the response surface methodology (RSM), the H_2_ production (mmol/L) and 1,3-propanediol (g/L) was chosen as the response variable. The interaction and relation between the input and the responses variables was determined by design matrix evaluation considering the significance *p*-value, *R*^2^ values for the models tested and final model equation in terms of factors was obtained from the analysis of variance (ANOVA) [2,38].

### 3.6. Comparative Study of Hydrogen Production across Pure-, Co- and Mixed-Culture

The optimized conditions of mixed-culture obtained from CCD model were used for a comparative study across pure culture of *Enterobacter aerogenes*, *Clostridium butyricum* and co-culture of these two bacteria with mixed-culture for H_2_ production. The anaerobic growth of *E. aerogenes* and *C. butyricum* were carried out in presence of basal media (minimal media) containing glucose (10 g/L), peptone (20 g/L), KH_2_PO_4_ (2 g/L), yeast extract (0.5 g/L), MgSO_4_·7H_2_O (0.5 g/L) and l-cysteine-HCl·H_2_O (1 g/L) included in case of *C. butyricum* [2,5,10]. The media preparation, sterilization and incubation were the same as explained in the previous section (H_2_ production using modified basal media).

### 3.7. Analytical Techniques

#### 3.7.1. Analysis of Hydrogen by GC

The collected gas sample using gas tight syringe in vacuumed sample vials at the end of fermentation was analyzed by gas chromatography (Agilent technology, Santa Clara, CA, USA) fitted with a 3 m PoraPLOT Q^®^ column (Agilent technology, Santa Clara, CA, USA) and equipped with a thermal conductivity detector (TCD). The GC set-up with injector, column temperature and detector temperature set at 100 °C and carrier gas nitrogen was used at a flow rate of 3.5 mL/min [10]. The volume of gas produced was calculated and converted to mmol, considering the temperature and atmospheric pressure during the experimental runs [38].

#### 3.7.2. Analysis of End-Metabolites/by-Products by GC-FID

The concentrations of 1,3-propanediol and glycerol were analysed on ZB-WAX plus column fitted with flame ionization detector (FID) detector in a gas chromatography (GC) (7890B GC-Agilent, Santa Clara, CA, USA) set-up. The GC condition at a flow rate of 1 mL/min using helium carrier gas at a temperature profile of 80–240 °C under 8.4 min method run time was developed [5].

## 4. Conclusions

The enrichment of H_2_ producing bacteria using five pretreatment methods (by acid, base, heat, chloroform and microwave respectively) were evaluated for H_2_ production from wastewater sludge using CG as substrate. The heat treatment resulted in eliminating H_2_ consuming bacteria with highest hydrogen production (15.18 ± 0.26 mmol/L) at 30 °C across the pretreatment methods. The CCD model was significant with (*p*-value: 0.028 and <0.0001) to identify the optimal CG concentration (20 g/L), inoculum size (20%) and pH (7) that positively influenced for increased H_2_ production (29.43 ± 0.71 mmol/L) with decreased by-product (1,3-PD: 5.12 ± 0.59 g/L) production. High correlation of pH, CG and InS resulted with model significant *p*-value of 0.028. The pH (*p*-value: 0.0011) had the highest impact on the H_2_ production in comparison to other parameters (InS and CG conc.). In the present study, under optimized conditions, the mixed-culture reported maximum H_2_ production and also provided efficient inoculum conditions in comparison to mono- and co-culture system. The heat pretreatment step used in this mixed-culture system is simple, cheap and industrially applicable in comparison to pure-/co-culture system for H_2_ production.
